# Validation of the Polish adaptation of the Students’ Punishment Sensitivity Scale in an educational context

**DOI:** 10.3389/fpsyg.2026.1850295

**Published:** 2026-08-21

**Authors:** Katarzyna Tomaszek, Agnieszka Muchacka-Cymerman

**Affiliations:** 1 Institute of Psychology, Uniwersytet Rzeszowski, Rzeszów, Poland; 2 Institute of Psychology, Humanitas University in Sosnowiec, Sosnowiec, Poland

**Keywords:** Poland, reliability, school burnout, student punishment sensitivity, validation

## Abstract

**Background:**

Reinforcement sensitivity theory highlights the crucial role of two systems: the behavioral inhibition system (BIS) and the behavioral activation system (BAS) in explaining one’s tendency to respond to positive or adverse stimuli in diverse contexts, including educational. However, there is a lack of measurements that capture the punishment mechanism underlying students’ reactions when confronted with others’ opinions on their school failures. The research project aimed to test the psychometric properties of the Polish version of the students’ punishment sensitivity (SPS-PL) in the educational context, originally developed for a Turkish sample.

**Methods:**

A sample of 548 students aged 12–15 years (53.1% boys) from randomly chosen public schools participated in the study. We used instruments that measure students’ punishment sensitivity (SPS-PL Scale) and school burnout (ESSBS). The factor structure (CFA), reliability, and validity analyses were performed. We also tested measurement invariance across gender.

**Results:**

CFA indicated acceptable goodness of fit indexes for the three-factor structure of the Students Punishment Sensitivity construct with domains such as: Inhibition due to punishment (IDP), Negative attitudes towards punishment contexts (NATPC), Regulatory Effect of Punishment (REP). The SPS-PL Total Score and IDP demonstrated acceptable reliability (*α*/*ɷ* over 0.80), while for NATCP and REP were *α* ≥ 0.67/*ɷ* ≥ 0.68. We evidenced its discriminant and convergent validity, i.e., the HTMT correlations over 0.30, and positive correlations between punishment sensitivity indicators and school burnout. A higher punishment sensitivity was related to poor quality of relationships with classmates, teachers, and parents. Full configural and metric invariance, a well as partial scalar and strict invariance across gender were confirmed. The findings also indicate that girls scores higher in SPS – PL Total score, IDP, and REP than boys.

**Conclusion:**

Studies on students’ punishment sensitivity in the context of school failure are scarce. At the same time, these adverse experiences may directly affect young people’s educational outcomes and seriously reduce their chances for flourishing development in the future. The research will enable scholars with a valid instrument that allows for a better understanding of this phenomenon.

## 1 Introduction

The manner in which a student responds to failure experienced in the school context, such as written or oral assessment, constitutes a key challenge for contemporary educational psychology. The challenge lies not in the “punishment” itself, but in how it is implemented within modern educational systems, so that students relate it solely to their behavior at a specific moment and within a particular subject, rather than generalizing it to themselves as learners and as individuals, extending this evaluation across all areas of their school life. Some research has focused on students’ motivational processes and academic achievement, however, comparatively little attention has been devoted to the mechanisms that shape students’ sensitivity to negative educational experiences (Lo et al., 2022; Natiqi, 2024). One such mechanism is their sensitivity to punishment (Slessareva and Muraven, 2004; Aypay, 2018a, 2018b).

### 1.1 Reinforcement sensitivity theory & punishment mechanism

Reinforcement Sensitivity Theory (RST) refers to biologically grounded mechanisms that regulate how individuals respond to rewarding and punishing stimuli (Corr, 2008). In its revised version, it distinguishes, among others, the Behavioral Activation System (BAS), associated with goal-directed behavior toward rewards, and the Behavioral Inhibition System (BIS), which is responsible for responding to signals of punishment, uncertainty, and goal conflict (Corr, 2008; Espinoza Oyarce et al., 2021). From this perspective, differences in the functioning of these systems may translate into how individuals experience and interpret situations involving evaluation, effort, or confrontation with the possibility of failure. Thus, sensitivity to punishment cannot be reduced solely to reactions to actual consequences; it also encompasses the ways in which potential threats are anticipated and interpreted. It is worth noting that school is a place where students experience repeated and multiple forms of assessment, and teacher-child interaction is seen as an important way for children to self-evaluate their learning abilities. As preadolescents navigate increasing cognitive and social demands, they tend to internalize teacher feedback—both verbal and nonverbal signals—using it to construct their academic self-concept, tailor their social interactions, and refine their study expectations (Wu and Zhang, 2022). However, punitive classroom management strategies may lead to poor mental wellbeing and class misbehaviours (Ijaz et al., 2024). As a result of such practices, students may exhibit a punishment oversensitivity, which leads to emotional disregulation, learning disengagement, maladaptive coping, and academic burnout (Aypay, 2018a; Erdogan et al., 2025; Tomaszek et al., 2025). Individuals with high punishment sensitivity tend to exhibit heightened vigilance, avoidance tendencies, and stronger emotional responses in evaluative contexts (Corr, 2008; Ridley, 2022; Kreuze et al., 2020). Similarly, students high in negative feedback sensitivity from teachers are at risk of academic fear and distress, react with disinterest when facing effortful tasks, but also often avoid interactions with fearful teachers through school refusal or truancy (Ishak, 2013; Aypay, 2015, 2018a; Lunetti et al., 2024). Based on Reinforcement Sensitivity Theory, punishment can therefore be understood as a regulatory system that not only inhibits behavior but also organizes the processing of information related to potential negative consequences. Contemporary approaches emphasize that responses to punishment result from complex interactions among emotional, cognitive, and social processes, rather than merely reflecting simple behavioral inhibition (Zhu et al., 2025; Jean-Richard-Dit-Bressel et al., 2018). An expansion of the above-mentioned framework is Sensitivity to Threat and Affiliative Reward (STAR) Model, which directly suggests that the level of threat sensitivity (cues of punishment) is a result of the interaction between genetic neural sensitivity and environments features (Waller and Wagner, 2019). Although the model is mainly focused on low level of punishment sensitivity as an antecedent of callous-unemotional traits developed by a child it was recently applied to an educational setting by Baroncelli et al. (2022). The results confirmed that the higher levels of sensitivity to teachers’ punishment is negatively related to callous-unemotional traits especially at low (versus high) levels of dissatisfaction with teachers.

When applied to the educational context, this perspective gains particular importance, as the punitive school practices may include direct forms of aggression (i.e., verbal scolding, physical slapping, restrict school rules, banning, public reprimand or threatening), but also more subtle forms such as a lack of support, over-criticism, passing over, poor teacher- student dialogue, internal exclusion, comparison with others or highlighting the role of skills not student efforts (Aypay, 2018a; Poudel, 2022; Jones et al., 2023; Douanla, 2025). Such adverse stimuli also include low grades or disapproval from teachers, parents, or peers, which have a distinctly social character and may be interpreted as signals of threat (DeVries et al., 2018; Kristensen et al., 2023). These harsh forms of school-related sanctions diminish student self-esteem, increase the feelings of shame, despair, loneliness, and anger, stifle intrinsic study motivation at the same time, yield school distress and burnout, but also directly hinder social–emotional development, by general oversensitivity to punishment context (Aypay, 2018a, 2018b; Jones et al., 2023). As stated by Eisler and Fry (2019), such conditions create punitive dominating approaches, that trigger the sympathetic part of the body’s autonomic nervous system, consequently putting individuals into a state of alert and decrease ability to learn. Worth adding that recently published theoretical framework connects student burnout with the repression-sensitization mechanism, which is directly linked to punishment vs. reward sensitivity. In regards, this construct is a type of regulatory system, a form of cognitive coping strategy employed when facing difficult and threatening events (Kleszczewska-Albińska, 2026). According to Weinberg, Schwartz, and Davidson, individuals may react as repressors (underestimation of one’s arousals) or sensitizers (overestimation of one’s adverse experiences) (Myers, 2010). More profoundly, these innate biological sensitivities, when reinforced by study demands, may block individuals’ adaptive reactions towards stressors, resulting in burnout (Kleszczewska- Albińska, 2026). In other words, sensitizers, who are highly reactive to threats, may exaggerate even small signals of discomfort, initiated by school duties or teachers instructions, searching for punishment signs. Such cognitive biases, accompanied by continued exposure to school stressors, may further intensify these maladaptive patterns, leading to the adoption of socially undesirable reactions in attempts to cope with overwhelming difficulties.

### 1.2 Students’ punishment sensitivity

Punishment sensitivity in educational settings refers to heightened reactivity to evaluative cues, such as criticism, low grades, or social disapproval. Empirical evidence suggests that students with elevated punishment sensitivity are more likely to exhibit avoidance-oriented behaviors, increased fear of evaluation, and lower academic engagement (Aypay, 2015; Corr, 2008). These findings indicate that punishment sensitivity may represent an important psychological mechanism influencing how students interpret and respond to evaluative experiences in school.

Previous research has shown that punishment sensitivity is a multidimensional construct comprising three core components: Inhibition Due to Punishment (IDP), Negative attitudes toward the punishment context (NATPC), and Regulatory Effect of Punishment (REP) (Aypay, 2015, 2018a, 2018b; see Table 1).

**Table 1:** The three core elements of the students’ punishment sensitivity construct developed by Aypay (2018a, 2018b).

Factor	Description
Inhibition due to punishment (IDP) – behavioral dimension	Strong belief in the adverse feedback of the school and family environment (classmates, teachers, parents) when facing educational failure after one’s answers during classes Strong anticipation of aggressive behaviours, such as being teased, humiliated, or otherwise punished for telling something wrong during classes Being passive and withdrawn from academic life; poor or lack of classroom participation due to a strong expectation of failing and being punished Fear of getting punished and trying to avoid punishment by not engaging in school activities
Negative attitudes towards punishment contexts (NATPC) – affective dimension	Various negative feelings that student develops towards school, teachers and courses due to experiencing punishment when trying to meet study demands The feeling of hatred of classes due to being punished by parents for school failures Negative attributions about teachers, school, and parents as an effect of punishment stimuli in the context of not meeting school demands
Regulatory effect of punishment (REP) – cognitive dimension	Mental strategies that allow individuals to avoid expected punishment in the academic context Negative Self-attributions due to being punished for one’s poor academic achievements, i.e., self-devaluation, self-anger, worthlessness

Cross-cultural research highlights that the role and expression of punishment sensitivity may vary across educational systems and socio-cultural contexts (Tomaszek et al., 2025; Peguero et al., 2021; Mulyati and Uyun, 2024). Importantly, according to *International Civic and Citizenship Education Study* (ICCS) report from 2022 year a majority of polish adolescents from eighth grade do not trust school institutions, with 39% of them declared not to feel they get along well with their teachers, which also includes the lack of right to freely express their own viewpoints during classes. Moreover, most respondents stated that polish school uses archaic learning strategies, mostly based on teachers authority and motivating students through tests and grades. Additionally, compared to other countries, nearly one in three Polish teenager pointed on poor teacher-students dialogue, being unfairly treated by teachers, as well as feeling not to be listened by them (IEA, 2022). Youth in Poland (N = 656) aged 13–17 years, declare not to be satisfied with the rules that exist in their school (40%), one in four think that school effectively hinders their plans fulfillment, and feel annoyed when they are unable to speak their mind in school matters that are important to them (55%) (Murawska, 2020). The abovementioned features of consequences for children polish educational system suggest the need for more in-depth analysis of punitive practices in schools and their’s well-being.

Research has also attempted to examine gender differences in punishment sensitivity; however, the findings remain inconclusive. Some studies suggest that girls may exhibit higher levels of this trait, which is often explained by greater emotional reactivity and heightened sensitivity to social evaluation (Aypay, 2015). Other findings indicate that punishment sensitivity is associated with stronger processing of negative feedback, and this effect persists even after controlling for gender, suggesting that gender differences do not fully account for this mechanism (Santesso et al., 2011). At the same time, research on the social acceptance of punishment indicates that gender influences how punitive behaviours are evaluated and justified: men tend to accept harsher forms of punishment more frequently, whereas women are more likely to consider the relational and normative context of a situation (Rajter and Medvidović, 2025; Dato and Friehe, 2025).

### 1.3 Punishment Sensitivity Scale

The Punishment Sensitivity Scale (SRS) used in the current study was developed by Aypay (2015) originally for Turkish student’s population. It is a self-report tool that contains 9 items falling into three subscales: inhibition due to punishment, negative attitudes towards punishment contexts, and Regulatory effect of punishment. It was created with secondary school students in mind and based on a Gray’s notion that “sensitivity to punishment, hinders individuals from achieving their goals and finalises the approach-hindrance tension mostly with an avoidance reaction, is responsible for feelings such as anxiety, fear, sadness and inhibition due to punishment, unrewardedness and innovation clues (Aypay, 2018b, p.2). SRS contains statements about avoidance of actions due to fear of punishment” (i.e, If I think I will get a negative reaction, I do not answer a question in class even though I know the answer), adverse emotional states caused by prior experiences of punishment (I hate the school classes when my parents punish me because I fail), and regulatory effect of punishment on self-evaluations (I get angry with myself when I perform a behaviour for which I get punished because of my classes). Respondents rate how each statement is suitable to his/her school experiences on a 4-point scale. The scale was validated on a large sample for middle school students (*N* = 32.688 subjects). In their original research, Aypay (2015) confirmed 3-factor structure of students’ sensitivity to punishment through Exploratory Factor Analysis showing 66.5% of the explained variances, and Confirmatory Factor Analysis (*χ*^2^ = 41.03, *df* = 24, *χ*^2^/*df* = 1.75; RMSEA = 0.05, SRMR = 0.05, CFI = 0.98) and found satisfactory internal consistency for SPS Total score *α* = 0.83 and for IDP *α* = 0.80, as well acceptable for NATCP *α* = 0.75, and for REP *α* = 61 (Aypay, 2015).

## 2 Current study aims and study hypothesis

The primary goal of the study was to examine the psychometric properties of the Polish version of the Students’ Punishment Sensitivity (SPS-PL).

We hypothesis that the CFA will support the three-factor structure of students’ punishment sensitivity (H1). We also aimed to examine the reliability and validity of the SPS-PL among Polish early adolescents. Reliability was assessed by evaluating the internal consistency of the total SPS-PL score and its three originally proposed subscales. Construct validity was examined by analyzing intercorrelations among the SPS-PL subscales and their associations with school burnout and the quality of social relationships. We hypothesized that the SPS-PL subscales would be positively correlated with one another. Furthermore, we expected higher punishment sensitivity to be associated with higher levels of school burnout. Finally, we hypothesized that punishment sensitivity would be negatively associated with the quality of relationships with classmates, parents, and teachers. Finally, we aimed to establish whether the structure of the punishment sensitivity reactions of young people shows measurement invariance across samples that differ in terms of gender. We hypothesize similar structure of the punishment sensitivity among boys and girls sample.

## 3 Materials and methods

### 3.1 Sample and procedure

Prior to data collection, the study protocol was approved by the University’s Ethics Committee (Resolution No. Dna.0046.1.13.2023). Data were collected between September 2022 and March 2023 in randomly selected public elementary schools located in different regions of Poland. Schools were selected from the official Register of Schools and Educational Institutions maintained by the Polish Ministry of National Education. Initially, 20 public elementary schools were randomly selected, and their principals received an email containing a description of the study and an invitation to participate. We estimated that the participation of at least 10 schools would be required to obtain a sample of more than 500 students. Following the schools’ agreement to participate, the formal consent procedure was initiated. This included providing information about the aims and procedures of the study to school principals, teachers, parents/legal guardians, and students, as well as explaining the paper-and-pencil method of data collection. The inclusion criteria were as follows: (i) written consent from school principals, teachers, and parents/legal guardians; (ii) enrollment in the seventh or eighth grade of a public elementary school; (iii) proficiency in the Polish language; and (iv) voluntary assent provided by the students. Data were collected using a paper-and-pencil questionnaire in accordance with the ethical principles of the Declaration of Helsinki. Before completing the questionnaires, students were informed that participation was voluntary, that all responses would remain anonymous, and that they could withdraw from the study at any time without any consequences. To ensure confidentiality, school staff had no access to the completed questionnaires or study documentation.

A total of 548 students completed the questionnaire battery. The sociodemographic characteristics of the sample are presented in Table 2.

**Table 2:** Sociodemographic characteristics of sample (*N* = 548).

Variable	Sex				Total (*n* = 548)	
	Girls (*n* = 257)		Boys (*n* = 291)			
Age M(SD)	13.18(0.67)		13.23(0.73)		13.21(0.70)	
	*n*	%	*n*	%	*n*	%
12 years	38	14.8	42	14.4	80	14.6
13 years	134	52.1	150	51.5	284	51.8
14 years	85	33.1	90	30.9	175	31.9
15 years	0	0.0	9	3.1	9	1.6
Student-teacher relationships						
Poor	31	12.1	65	22.3	96	17.5
Average	151	58.8	144	49.5	295	53.8
Good	75	29.2	82	28.2	157	28.6
Student-parent relationships						
Poor	6	2.3	19	6.5	25	4.6
Average	75	29.2	45	15.5	120	21.9
Good	176	68.5	227	78.0	403	73.5
Student–student relationships						
Poor	32	12.5	16	5.5	48	8.8
Average	120	46.7	62	21.3	182	33.2
Good	105	40.8	213	73.2	318	58.0

### 3.2 Instruments

#### 3.2.1 Translation process – preliminary development of SPS-PL version

First, permission to adapt the School Punishment Sensitivity (SPS) Scale for use in the Polish population was obtained from the author of the original instrument (Aypay, 2015). The English version of the nine-item SPS, provided by the author of the original Turkish scale, was translated into Polish by a bilingual psychologist. The translated version was then back-translated into English by a native English speaker employed by a professional translation company. The back-translated version was independently compared with the original instrument by two psychologists, and no discrepancies were identified. The Polish version of the SPS (SPS-PL) was subsequently reviewed by two experts from different research centers with expertise in educational psychology and psychometrics. They evaluated the semantic equivalence, clarity, and comprehensibility of the items. The experts concluded that the item content reflected common experiences in both school and family contexts and could therefore be considered culturally universal and appropriate for Polish adolescents. As no further revisions were recommended, this version was accepted as the final SPS-PL and used in the present study. During data collection, none of the participants reported difficulties in understanding or completing the questionnaire.

#### 3.2.2 The Study Punishment Sensitivity Scale (SPS-PL)

The SPS-PL scale was designed in Turkey by Aypay (2015, 2018a, 2018b) and considered a short tool (9 items) that determines middle school students’ reactions to punishment received due to school failures from important others (classmates, parents, teachers). The instrument consists of three subscales: Inhibition due to punishment (IDP, 3 items), Negative attitudes towards punishment contexts (NATPC, 3 items), and Regulatory Effect of Punishment (REP, 3 items) (i.e., *I hate the school classes when my parents punish me because I fail*). The Likert-type response format with four response options is used: 1 – “Strongly agree,” 4 – “Strongly disagree.” No reverse items are included in the scale. The Polish version of SPS retained the original scoring direction: the score for each subscale is the sum of the items that comprise it; the overall score is the sum of all 9 items. The higher SPS-PL score the higher level of punishment sensitivity. The documented reliability of Cronbach’s *α* in the original study sample for the total score was equal to 0.80, and for subscales ranged between 0.61 and 0.80 (Aypay, 2015, 2018b).

#### 3.2.3 The Elementary Student School Burnout Scale (ESSBS)

The ESSBS by Aypay, in Polish adaptation by Tomaszek and Muchacka-Cymerman (2019a), was used to measure study burnout among elementary school pupils. The instrument comprises 24 items rated on a 4-point Likert scale: 1 – “Strongly agree,” 4 – “Strongly disagree” (e.g., “*School is tiring for me*”). The psychometric properties of the ESSBS-PL have been confirmed in several studies, consistently showing good reliability (Tomaszek and Muchacka-Cymerman, 2019a, 2019b; Tomaszek, 2020). In this study, internal consistency was high for the total score (*α*/*ɷ* = 0.90) and good for the subscales (*α*/*ɷ* ranged from 0.76 to 0.84).

### 3.3 Data analysis

SPSS 22.0 and Jamovi 2.3.28were used for data processing and statistical analysis. No missing data were detected in the data. Through preliminary analysis, we detected non-normal multivariate data distribution; Mardia’s kurtosis = 117.8, *p <* 0.001, and Mardia’s skewness = 11.1, *p <* 0.001. Therefore, we calculated Confirmatory Factor Analysis (CFA), we used Robust Weighted Least Squares (WLSM) method of estimation with Satorra-Bentler mean adjusting. The following fit index criteria were used to establish models fit: CFI with a value higher than 0.900, RMSEA with a value lower than 0.08, and SRMR with a value lower than 0.08 (Bentler and Bonett, 1980; Bentler, 1999; Chen, 2007). To check the reliability of the SBS-PL, Composite Reliability, as well as Cronbach’s α and McDonald’s *ɷ* data were estimated. In the next step, we calculated convergent and discriminant validity by applying Composite SEM module from Jamovi and calculating: Heterotrait-Monotrait Ratio of Correlations (HTMT) and Average Variance Extracted (AVE). We also checked the associations between SPS-PL scores and ESSBS indicators (Pearson’s coefficients), as well as between SPS-PL and social relationships with important others (Spearman’s analysis). Measurement invariance of the SPS-PL was examined according to gender differences in punishment sensitivity structure across the total sample with such criteria as: for metric invariance: ΔCFI≤ 0.010, ΔRMSEA ≤ 0.015, and ΔSRMR ≤ 0.030; for residual invariance similar criteria for changes in CFI and RMSEA and for ΔSRMR ≤ 0.010 (Chen, 2007). We also presented scores for changes in *χ*^2^, however, we decided not to guide by this criterion in decisions regarding the invariance analysis due to its biased outcomes causing by its strong sensitivity on sample sizes equal to or larger than 300 (Chen, 2007).

## 4 Results

### 4.1 Confirmatory factor analyses

The CFA results indicated statistically significant chi-square tests for all tested models. However, Models 1a and 2a demonstrated poor fit, particularly with respect to the RMSEA and SRMR indices, which did not meet the recommended threshold of 0.08. After examining the modification indices and the content of the items, we added a covariance between Items 5 and 6. Both items load on the same subscale and reflect closely related content, referring to dislike of school (Item 5) or teachers (Item 6) as a consequence of experiencing punishment. Model 2b demonstrated an acceptable fit to the data, meeting the recommended criteria (*χ*^2^/*df* ≤ 5.0, CFI ≥ 0.90, RMSEA < 0.08, and SRMR < 0.08; see Table 3). In addition, all standardized factor loadings exceeded the recommended value of 0.40 (see Table 4).

**Table 3:** CFA statistic fit indexes (*n* = 548).

Number of model	*χ* ^2^	*df*	*p*	CFI	RMSEA (90% CI)	SRMR	TLI	GFI
1a. 1- factor model	307	27	<0.001	0.878	0.141 [0.131; 0.151]	0.105	0.837	0.990
1b. 1- factor model	134	26	<0.001	0.953	0.092 [0.082; 0.102]	0.061	0.935	0.996
2a. 3-factor model	131	24	<0.001	0.953	0.095 [0.085–0.106]	0.073	0.930	0.996
2b. 3-factor model	51.1	23	<0.001	0.988	0.057 [0.047; 0.068]	0.047	0.981	0.998

Robust results were reported; Model 1a;2a: no covariance between items; Model 1b;2b: Covariance between items: 5–6 represent similar content in the same subscale.

**Table 4:** Factor loadings in CFA.

Item Nb.	Model 1b (univariate structure)					Model 2b (3-factor structure)					
	*β*	SE	*p*	95% CI		Factor	*β*	SE	*p*	95% CI	
				Lower	Upper					Lower	Upper
Item1	0.72	0.00	<0.001	0.67	0.77	IDP	0.80	0.00	<0.001	0.75	0.85
Item2	0.76	0.06	<0.001	0.70	0.81		0.84	0.06	<0.001	0.78	0.89
Item3	0.65	0.05	<0.001	0.60	0.71		0.71	0.05	<0.001	0.66	0.77
Item 5	0.39	0.06	<0.001	0.30	0.48	NATPC	0.76	0.00	<0.001	0.68	0.84
Item 6	0.23	0.06	<0.001	0.14	0.32		0.60	0.07	<0.001	0.51	0.69
Item 7	0.51	0.06	<0.001	0.44	0.59		0.65	0.98	<0.001	0.57	0.74
Item 4	0.55	0.06	<0.001	0.48	0.62	REP	0.56	0.00	<0.001	0.48	0.64
Item 8	0.59	0.06	<0.001	0.52	0.66		0.63	0.11	<0.001	0.56	0.69
Item 9	0.68	0.06	<0.001	0.62	0.74		0.73	0.12	<0.001	0.66	0.79

IDP, inhibition due to punishment; NATPC, negative attitudes towards punishment contexts; REP, regulatory effect of punishment.

### 4.2 Reliability

The total scores of the Polish version of the SPS-PL and IDP scales demonstrated good internal consistency (*α* = 0.817, *ω* = 0.824, and *α* = 0.825, *ω* = 0.828, respectively). The NATPC and REP subscales showed acceptable reliability (NATPC: *α* = 0.694, *ω* = 0.753; REP: *α* = 0.667, *ω* = 0.684). Item-level reliability statistics are presented in Table 5.

**Table 5:** Item reliability statistics.

Nb of item	Mean	SD	Item-rest correlation	Skewness	SE	Kurtosis	SE	If item dropped	
								Cronbach’s *α*	McDonald’s *ω*
Item 1	2.52	1.05	0.592	0.02	0.10	−1.18	0.21	0.790	0.793
Item 2	2.22	1.01	0.623	0.43	0.10	−0.87	0.21	0.786	0.789
Item 3	2.21	1.09	0.520	0.41	0.10	−1.12	0.21	0.799	0.801
Item 4	2.00	1.00	0.494	0.69	0.10	−0.63	0.21	0.802	0.807
Item 5	2.66	1.06	0.461	−0.17	0.10	−1.19	0.21	0.806	0.813
Item 6	2.41	0.98	0.328	0.25	0.10	−0.94	0.21	0.821	0.825
Item 7	1.95	1.00	0.499	0.81	0.10	−0.41	0.21	0.801	0.808
Item 8	2.21	0.99	0.528	0.30	0.10	−0.98	0.21	0.798	0.802
Item 9	2.06	1.00	0.604	0.51	0.10	−0.90	0.21	0.788	0.793

Response distribution in each item ranged from 1 to 4.

Separate analyses by sex indicated similar internal consistency for the total SPS-PL score among girls and boys (*α* = 0.828 and *α* = 0.806, respectively). Reliability coefficients for the NATPC subscale were *α* = 0.645 for girls and *α* = 0.730 for boys. In contrast, girls demonstrated higher reliability for the IDP scale (*α* = 0.852 vs. *α* = 0.759), whereas boys showed higher reliability for the REP subscale (*α* = 0.739 vs. *α* = 0.598).

### 4.3 Discriminant and convergent validity

The established discriminant validity through the Heterotrait-Monotrait Ratio of Correlations (HTMT) were less than the strict thresholds (<0.85, Darwisman, 2025) indicating that all three SBI subconstructs are distinct (see Table 6).

**Table 6:** The SPS-PL scale discriminant validity.

Latent variables intercorrelations	HTMT1	HTMT2
IDP - Latent1 ↔ NATPC - Latent2	0.366	0.322
IDP - Latent1 ↔ REP - Latent3	0.774	0.772
NATCP - Latent2 ↔ REP - Latent3	0.669	0.625

HTMT1, original HTMTm, a*rithmetic mean* of the correlations of items; HTMT2, improved HTMT, *geometric mean* of correlations of items; IDP, inhibition due to punishment; NATPC, negative attitudes towards punishment contexts; REP, regulatory effect of punishment.

Convergent validity was assessed using the Average Variance Extracted (AVE) and Composite Reliability (CR). The AVE values exceeded the recommended threshold of 0.50 for the IDP and NATPC subscales but fell below this criterion for the REP subscale. However, the CR values for all subscales exceeded the recommended threshold of 0.70, indicating acceptable convergent validity despite the lower AVE observed for the REP subscale (Fornell and Larcker, 1981) (see Table 7).

**Table 7:** The SPS-PL scale convergent validity and reliability.

	Variable	Cronbach’s alpha (*α*)	McDonald’s (*ω*)	CR Dijkstra-Henseler’s rho (*ρ*A)	AVE
Model 2b	IDP - Latent1	0.825	0.828	0.833	0.617
	NATCP - Latent2	0.694	0.753	0.850	0.524
	REP - Latent3	0.667	0.684	0.716	0.426

CR, composite reliability; AVE, average variance extracted; IDP, inhibition due to punishment; NATPC, negative attitudes towards punishment contexts, REP, regulatory effect of punishment.

All Students’ Punishment Sensitivity indicators correlated positively with school burnout and negatively with self-efficacy, confirming SPS-PL convergent validity (see Table 8).

**Table 8:** Correlation analysis between tested variables.

Variables	M (SD)	Min.–max.	1	2	3	4	5
1. IDP	6.96 (2.70)	3–12	—				
2. NATPC	7.06 (2.25)	3–12	0.33^***^	—			
3. REP	6.22 (2.27)	3–12	0.56^***^	0.58^***^	—		
4. SPS-PL	20.23 (5.84)	9–36	0.80^***^	0.76^***^	0.87^***^	—	
5. ESSBS	67.22 (12.93)	32–104	0.41^***^	0.64^***^	0.53^***^	0.64^***^	—

*p < 0.05; **p < 0.01; ***p < 0.001.

IDP, inhibition due to punishment; NATPC, negative attitudes towards punishment contexts; REP, regulatory effect of punishment; SPS-PL, student punishment sensitivity total score; ESSBS, school burnout.

The convergent validity analysis of the SPS-PL scale also included testing for correlations between quality of relationships with important others (i.e., classmates, teachers, and parents). We detected negative correlations between SPS-PL and all examined types of bonds (rho ranged from −0.18 to −0.38, *p* < 0.001). Additionally, IDP and REP also correlated negatively with classmates, teachers and parents quality of relationships (rho ranged from −0.12, *p* < 0.01 to −0.38, *p* < 0.001), while the coefficients for NATPC were significant and negative for teachers and parents bonds (rho = −0.33, and *r* = −0.32, *p* < 0.001, respectively), and insignificant for classmates (rho = 0.03, *p* = 0.422).

### 4.4 Measurement invariance across gender

The results of testing the 3-factor structure equivalence of punishment sensitivity for gender were conducted using the four invariance indices: configural, metric, scalar, and residual. The three-factor structure had an acceptable goodness of fit index in all invariance models.

*Configural invariance*: the baseline multi-group model demonstrated an adequate fit across gender groups (*χ*^2^ = 89.90, *p* < 0.001, CFI = 0.981, RMSEA = 0.074, SRMR = 0.061). These findings indicate that the three-factor structure and the pattern of item loadings were equivalent across girls and boys.

*Metric invariance*: the chi-square difference test did not support full equality of factor loadings across groups (Δ*χ*^2^, *p* < 0.01). However, because the chi-square statistic is highly sensitive to sample size, it may indicate significant differences even when practical differences are negligible. Therefore, following the recommendations of Chen (2007), we evaluated changes in CFI, RMSEA, and SRMR. The results supported metric invariance, as all changes in fit indices met the recommended criteria (ΔCFI ≤ 0.010, ΔRMSEA ≤ 0.015, and ΔSRMR ≤ 0.030; Chen, 2007; Putnick and Bornstein, 2016; see Table 9). Nevertheless, item-level constraint tests revealed one significant difference in the loading of item 9 (*χ*^2^ = 4.056, *p* = 0.033).

**Table 9:** Multi-group measurement invariance models comparison (girls *n* = 257) vs. boys (*n* = 291).

Type of MI	*χ* ^2^	df	∆*χ*^2^	RMSEA	∆RMSEA	SRMR	∆SRMR	CFI	∆CFI
1. Configural	89.9	46	—	0.074	—	0.061	—	0.981	—
2. Metric	115	52	25.1^***^	0.078	0.004	0.069	0.008	0.973	0.008
3a. Scalar	143	58	28^***^	0.084	0.006	0.075	0.006	0.964	0.009
4a. Residual	162	67	19^*^	0.082	0.002	0.080	0.005	0.960	0.004
3b. Partial Scalar invariance	120	54	5	0.079	0.005	0.069	0.000	0.972	0.008
4b. Partial Residual Invariance	143	63	23^**^	0.080	0.001	0.075	0.006	0.966	0.006

*p < 0.05; **p < 0.01; ***p < 0.001.

Robust results are reported.

*Scalar invariance*: the scalar invariance model demonstrated acceptable CFI and SRMR values, whereas the RMSEA was slightly above the recommended cutoff (0.08). Changes in the fit indices (ΔCFI = 0.009, ΔRMSEA = 0.006, and ΔSRMR = 0.005) nevertheless supported meaningful comparisons of latent means across gender groups. Item-level constraint tests indicated partial scalar invariance. Significant differences were observed for both the loading (*χ*^2^ = 7.03, *p* = 0.008) and intercept (*χ*^2^ = 7.66, *p* = 0.006) of Item 2, as well as for the intercepts of Item 4 (*χ*^2^ = 9.60, *p* = 0.002), Item 8 (*χ*^2^ = 12.17, *p* < 0.001), and Item 9 (*χ*^2^ = 10.47, *p* = 0.001).

Further analyses revealed statistically significant differences in latent means for two constructs. Compared with the reference group (boys), girls scored significantly higher on the IDP latent factor (estimate = −7.66, *p* < 0.001) and the REP latent factor (estimate = −2.54, *p* = 0.011), suggesting gender differences in these two dimensions of punishment sensitivity.

Therefore, a partial scalar invariance model was estimated by releasing the equality constraints for the loadings and intercepts identified above. Although model fit improved, the RMSEA remained slightly above the recommended threshold (CFI = 0.971, TLI = 0.961, RMSEA = 0.081, SRMR = 0.069). Consequently, the residual covariance of the NATPC factor was freely estimated, resulting in an acceptable model fit (CFI = 0.972, TLI = 0.962, RMSEA = 0.079, SRMR = 0.069).

*Strict invariance*: the initial strict invariance model, which constrained factor loadings, intercepts, residual variances, and latent factor variances to equality across groups, demonstrated acceptable fit on all indices except RMSEA (0.082). A partial strict invariance model was therefore estimated, yielding acceptable fit across all indices, including RMSEA (0.080). Furthermore, changes in CFI, RMSEA, and SRMR supported strict measurement invariance between girls and boys.

### 4.5 Gender differences

Gender differences were examined using Welch’s *t*-test because the assumption of homogeneity of variances was violated for the IDP and REP scales, as indicated by Levene’s tests (*F* = 11.95, *p* < 0.001, and *F* = 4.27, *p* = 0.039, respectively). According to the results girls scored significantly higher in IDP, REP, and SPS-PL Total score, while the findings for NATPC were insignificant (see Table 10).

**Table 10:** Differences in punishment sensitivity construct (observable and latent factors) across participants’ sex.

Variable	Observed		*t* welch	*p*	Effect size *d*	Latent mean difference	*z*	*p*
	Girls M (SD)	Boys M (SD)						
IDP	7.88 (2.70)	6.14 (2.42)	7.89	<0.001	0.68	−0.57	−7.66	<0.001
NATCP	7.07 (2.18)	7.05 (2.32)	0.13	0.894	0.01	0.010	0.16	0.876
REP	6.46 (2.38)	6.00 (2.14)	2.36	0.019	0.20	−0.16	−2.54	0.011
SPS-PL total	21.41 (5.85)	19.19 (5.65)	4.51	<0.001	0.39	—	—	—

IDP, inhibition due to punishment; NATPC, negative attitudes towards punishment contexts; REP, regulatory effect of punishment; SPS-PL, student punishment sensitivity total score.

## 5 Discussion

The current research aim was to psychometrically evaluate the Punishment Sensitivity construct measured with the Polish version of Students’ Punishment Sensitivity in Educational Context (SPS-PL).

### 5.1 Factor structure, reliability, and validity of SPS-PL

Based on the CFA method, we confirmed the three-factor structure of the examined SPS-PL scale, similar to the original construct developed by Aypay (2015) among Turkish students. According to the results, Polish early adolescents may inhibit their reactions in the classroom/home due to subjective signs of their colleagues’/teachers’/parents’ disapproval (IDP). They also experience negative attitudes toward school (i.e., dislike of situations or places) due to adverse beliefs of expected punishment (NATPC). Finally, external control of their reactions when confronted with failure was observed (REP), which also reflects the modulation effect of the cognitive processing of anticipated negative feedback. However, it is worth noticing that we observed smaller loading for one item than originally (item 6 in 1-factor model), which may suggest the lower ability to differentiate respondents based on the abovementioned item. Importantly, the lower loading of this item may also stem from the issue that is estimated. Particularly, it refers to teachers’ punishment practices, such as poor grades or negative comments when the student does not know the correct answer. Considering that teachers’ punishment may take multiple forms, i.e., scolding, slapping, disguising, threatening, and a lack of advising when students need support (Poudel, 2022), this item may be too general in its content. On the other hand, low grades may be the result of students’ poor knowledge objectively rated, or their insincerity—cheating, misbehaviours (lack of focusing on assigned tasks, incivility), and the demonstration of disinterest in the subject – aversion to learning manifested by using phones during classes or interrupting teachers due to feel bored or irritable (Guo, 2024). Moreover, these reasons may not be perceived by students as teachers’ retaliatory reactions but rather as strategies to motivate or discipline the classroom. What is more, this item also refers to liking vs. disliking teachers for the way of introducing discipline or motivating pupils. However, youth, due to adolescence developmental period, may dislike or disrespect all teachers in general, which may be a sign of the identity formation stage, a higher criticism and egocentrism, or conformity to group authorities (Ibrahim and Zaatari, 2020). In addition, prior studies have revealed that the young teenage students mostly express liking their teachers when the teachers were emotionally positive and socially accommodating (John et al., 2018), which suggests that the attitude toward teachers is a more complex phenomenon, and is not based only on the youth’s perception of teaching practices, as highlighted in item 6. These effects may directly impact our results, because we only measured students from 7th and 8th grade, while not capturing those from 6th grade perception, but also reported difficulties may refer to this process, not teachers’ reactions per se. In summary, a revision and extension of this item regarding multiple forms of teachers’ punishments, and not using the term liking but rather asking for the opinion on teaching practices, is recommended.

### 5.2 Measurment invariance across gender

We also showed SPS-PL capability to be used among individuals that differ in terms of their gender. The examined method presents a comparable structure across the populations of boys and girls (metric invariance), however, we observed partial scalar and strict invariance. Notably, our findings suggest that girls tend to score significantly higher in SPS-PL total score, and its two factors (i.e., IDP, REP) than boys. The observed effect is aligned with prior research in personality and neuroscience, which consistently presents females as exhibiting a higher sensitivity to punishment compared to males (Unger et al., 2012; Eneva et al., 2017; Dhingra et al., 2021). Such gender differences are strongly related to neural mechanisms underlying reward and punishment stimuli, with males presenting a higher response of ventral striatum to monetary reward and females to both monetary and social rewards (Li et al., 2023). It is also worth adding that gender discrepancies are associated with cultural norms regarding reactions to failure. More specifically, research has acknowledged that women are more prone to internalizing failures and frustrations, with a tendency to attribute its causes to a lack of proper abilities. In contrast, men externalize the sources of it more frequently and credit others or bad luck (Li et al., 2023). Such patterns of reactions are observed even at early stages of education, among preschool children, when boys tend to react on problems through withdrawn syndrome, while girls scored suffered from somatic complaint syndrome (Maldonado-Martinez et al., 2025). Importantly, some scholars argue that these differences may be an example of a gender-paradox in which boys perform and feel better than girls in countries where gender-equality norms are higher (Eriksson and Strimling, 2023). When considering the educational context, punishment oversensitivity may illustrate a more general pattern of failure reactions and school disengagement processes. Given that prior studies have shown that female teenagers are more likely to rely on teacher support (Burns et al., 2019) with an increased tendency to give up and self-handicap after the transition to secondary school (Yu et al., 2021), young girls may suffer more when facing punishment tactics implemented to discipline and control the classroom environment. Moreover, because fear of failure was found to be associated with lower social and emotional well-being (Borgonovi and Han, 2021), girls may develop a false impressions regarding school demands management and constitute a self-devaluation patterns of believes (i.e., I am not good enough) (Elliot and Thrash, 2004), resulting in ineffective educational strategies (lowered effort, lower persistence, and poorer performance) and deceptive decisions when thinking about future academic successes to minimise the possibility of punishment related to failing (Borgonovi and Han, 2021).

### 5.3 SBS-PL scale reliability

Our findings suggest acceptable reliability of the SPS-PL Total score and IDP scale *α*/*ɷ* over 0.80 for Total score, as well as marginal for NATCP and REP (*α*/*ɷ* equal or above 0.67). Nevertheless, a further reliability analysis conducted separately for girls and boys revealed that the result for boys sample in REP is equal to *α* = 0.598. Despite the fact, that according to some researchers, the validity of Cronbach’s alpha coefficient is closely dependent on the number of items included in a given scale may be lower due to the small number of test items (it consists of only 3 items), alpha values in the range of 0.45–0.60 are considered acceptable in such cases (Bretz and McClary, 2014; Taber, 2018), we believe that the observed lower coefficient needs further explanation, and should be taking into accounts by practitioners interested in using this scale. The REP scale includes two items that are focused on negative self-judgments when confronted with school failure, while the third one refers to an avoidance strategy as a way of coping with expected failure. This discrepancy may activate self – protection mechanisms different regarding students’ sex, but also caused by the level of public disclosure (the punishment used by teachers in front of classmates (item 6), adverse reactions of parents, which is more private (item 5), and negative self-evaluations and related emotions (hatred of classes) (item 7). It is worth adding that failure as a performance-outcome is avoided because it triggers adverse emotional states, such as shame and embarrassment, which affects global-self (Atkinson, 1964). In addition, numerous of prior research have confirmed sex differences regarding reacting on failure among secondary school students. In general, females tend to experience a higher fear of failure and its overgeneralization (McGregor and Elliot, 2005). In this regards, Caplan and Kinsbourne (1974) found that failing girls tend to pleasing the teacher through good behaviour and by this strategy increase their self-esteem, whereas most failing boys restore self-image by focusing on other than academic ways of experience success, i.e., being excellent in sports or leadership, but at the same time they tend to behave antisocially, which further discredits them with the teachers’ eyes, and setting up a vicious cycle. The abovementioned gender discrepancies may causes differences in reactions, leading to poorer internal consistency among boys group.

### 5.4 SBS – PL scale validity

The discriminant validity of the SPS-PL 3-factors was confirmed by calculating the HTMT correlations, which ranged from 0.32 to 0.77. These results are within the recommended threshold of a maximum of 0.85 (Ringle and Sarstedt, 2015). We also estimated the convergent validity by testing the significance of the relationships between SPS – PL indicators and school burnout. As expected, we observed positive correlations with school burnout syndrome, which is consistent with prior theoretical and empirical findings (Aypay, 2015, 2018a, 2018b). We also found a negative relationship between SPS-PL indicators and the quality of relationships with important others (i.e., classmates, teachers, and parents). This result is consistent with the tenet that punishment sensitivity is a mechanism that shapes social relationships and behavioral patterns of interactions with important others (Telzer et al., 2022). As a self-protecting response individuals present as a higher tendency to attain someone’s approval, as well as to detect and avoid social disapproval and its negative consequences like criticism, rejection, or social failure (Clark et al., 2015; Gu et al., 2020), hence it may increase conformity to peer group and a fear of others’ reactions, which directly negatively affects quality of relationships (Telzer et al., 2022). Interestingly, the results for NATPC and peer relationships was insignificant, which needs some further explanation. All items from this subscale refer to negative attitude toward punishment contexts, that only encompasses parents/school or teachers reactions on students’ poor achievements. Nevertheless, the fear of social exclusion and disapproval may also be related to classmates opinions, thus future revisions of the validated SPS-PL tool should consider extending the content of NATPC. On the contrary, in adolescence peers are rather a source of support, and build a union front against adults reality and priorities, which may be the rationale for narrowing the attributions made by students to parents or teachers.

### 5.5 Future directions and study limitations

The current project represents valid data for theoretical and practical contributions significant for the structure of major adversity - punishment in the educational context for failure in school achievements, faced by most students. The instruments allow for quick detection of those from the academic community who, due to expectations of adverse others’ reactions, inhibit school activities and thus experience lower outcomes than their actual cognitive potential. School counselors, through applying the punishment sensitivity scale in their everyday practices or research projects, may evaluate these difficulties and develop effective programs to minimize their adverse impact on the psychosocial and educational functioning of young people. This tool may also serve as a source of information on the impact of students’ sensitivity to punishment on their developmental trajectories.

Despite valuable contributions mentioned above, we must recognize some limitations of the current project that may impact its generalization. First, the data were collected only among students from 7th and 8th Grades in Elementary Schools, which limits the generalization of the results to (1) all early adolescent populations; (2) other educational levels in Poland. The level of punishment sensitivity in general may also be biased, due to the self-report method of data collection. To expand the Polish SPS-PL in early adolescents’ populations, future research projects should explore samples from all types of educational institutions (private, public, catholic; integrated, inclusive); include those with a higher vulnerability on punishment sensitivity in educational context (i.e., excluded individuals, or more distressed pupils); implement a more objective way of punishment sensitivity evaluation, (i.e., judged by teachers, parents); develop a voluntary program for further revision and refinement of the SPS-PL. Finally, we recommend an intercultural survey projects that allow for testing the universal and unique patterns in educational systems (e.g., grading culture, teacher-student relational norms), which potentially may moderate the associations between punishment sensitivity and its consequences.

## 6 Conclusion

The present study advances both measurement and theory by providing robust evidence for the validity of the Polish adaptation of the Students’ Punishment Sensitivity Scale (SPS-PL) within an educational context. The confirmation of its three-factor structure, satisfactory reliability, and measurement invariance across gender supports the conceptual stability of punishment sensitivity as a multidimensional construct in early adolescence. Our findings extend beyond psychometric validation by demonstrating that punishment sensitivity constitutes a meaningful psychological mechanism underlying students’ academic and socio-emotional functioning. The observed pattern of associations, whcih has positive links with school burnout and negative links with self-efficacy and relationship quality, suggests that heightened sensitivity to punishment may operate as a vulnerability factor, shaping maladaptive responses to evaluative and failure-related experiences in school environments. Importantly, the findings challenge simplified assumptions about the effectiveness of punishment as a regulatory tool in educational practice. Rather than promoting adaptive adjustment, heightened punishment sensitivity appears to be associated with avoidance-oriented strategies, diminished engagement, and relational difficulties. This underscores the need to reconsider the role of punitive practices in schooling, particularly in light of their potential to exacerbate vulnerability among already at-risk students.

From an applied perspective, the SPS-PL offers a theoretically grounded and empirically validated tool for identifying students who may be particularly susceptible to the adverse effects of evaluative stress and negative feedback. Its integration into school-based assessment frameworks may facilitate early detection and inform interventions targeting self-regulation, resilience, and adaptive coping.

## Data Availability

The dataset used in this study is not publicly available due to ethical and legal restrictions related to the protection of minors’ personal data. Participants were aged 12–15, and data collection was conducted in accordance with institutional ethical guidelines and parental consent procedures, which limit data sharing. Anonymized data may be made available from the corresponding author upon reasonable request and subject to approval by the relevant ethics committee. Requests to access these datasets should be directed to ktomaszek@ur.edu.pl.
